# Evaluation of autologous fat injection as a minimally invasive technique for rectocele repair: a prospective pilot study

**DOI:** 10.1007/s10151-026-03322-8

**Published:** 2026-05-01

**Authors:** I. A. Shafik, M. M. Abdelfatah, A. Shafik, E. Hagagy, M. Ali

**Affiliations:** 1 Department of General Surgery, Faculty of Medicine, Cairo University, Cairo, Egypt; 2 Department of Surgery, School of Medicine, New Giza University (NGU), Cairo, Egypt; 3 Department of General Surgery, Ahmed Maher Teaching Hospital, Cairo, Egypt

**Keywords:** Rectocele, Fat grafting, Obstructed defecation, Regenerative surgery, Minimally invasive, Pelvic floor repair

## Abstract

**Background:**

Rectocele repair remains challenging owing to high recurrence and morbidity associated with traditional surgical techniques. Autologous fat injection has emerged as a biologically integrative method that strengthens native pelvic tissues through regenerative mechanisms. This prospective pilot study evaluated feasibility, safety, and short-term anatomical and functional outcomes of autologous fat injection for rectocele repair.

**Methods:**

Between October 2023 and June 2025, 25 women with symptomatic Baden–Walker grade II–III rectocele and obstructed defecation syndrome (ODS) were prospectively enrolled at Kasr Al-Ainy Hospital, Cairo University. Abdominal fat was harvested, mechanically emulsified, and injected into the rectovaginal septum using a 15-gauge blunt cannula (mean injected volume 52 ± 10 mL). Outcomes included rectocele size on magnetic resonance (MR) defecography, clinical Baden–Walker grade, predefined ODS symptom categories/defecation frequency, and complications.

**Results:**

Mean rectocele size decreased from 3.28 ± 0.82 to 1.82 ± 0.47 cm (*p* < 0.001). Overall, 80% of patients reported complete symptomatic improvement, while 20% reported recurrent symptoms during follow-up. Transient postoperative constipation occurred in 20% of patients and resolved conservatively. No major complications occurred. Injected volume showed an exploratory association with anatomical improvement.

**Conclusions:**

Autologous fat injection for rectocele repair appears feasible and safe, with a signal of short-term anatomical and functional improvement in this pilot cohort. Larger comparative studies using validated patient-reported outcomes and standardized imaging follow-up are required.

## Introduction

Pelvic floor disorders represent a major health burden, particularly among multiparous and postmenopausal women. Posterior compartment defects such as rectocele commonly cause obstructed defecation syndrome (ODS), characterized by straining, digitation, incomplete evacuation, and perineal support. The lifetime risk of surgery for pelvic organ prolapse (POP) in women exceeds 11%, with posterior compartment defects constituting a significant proportion of these cases [1].

Traditional surgical options for isolated posterior compartment prolapse—such as posterior colporrhaphy or site-specific rectocele repair—are associated with variable anatomical and functional outcomes. Abramov et al. reported a recurrence rate of 33% following site-specific repair compared with 14% after standard posterior colporrhaphy [2]. Similarly, a prospective registry comparing posterior colporrhaphy and nonabsorbable mesh reinforcement for recurrent rectocele demonstrated higher anatomical cure rates with mesh but at the expense of increased mesh-related complications [3]. These findings highlight ongoing uncertainty regarding the optimal surgical approach for rectocele repair [4].

Minimally invasive alternatives such as stapled transanal rectal resection (STARR) have demonstrated symptomatic improvement in selected patients with ODS; however, recurrence, urgency, and procedure-specific morbidity remain concerns. Comparative studies have shown that while STARR may provide greater relief of obstructive symptoms, overall complication rates are comparable to posterior compartment repair [5]. Collectively, these data underscore continued interest in alternative strategies aimed at improving posterior compartment support while minimizing morbidity.

Parallel to these surgical developments, regenerative and biologically integrative approaches have gained attention. Adipose tissue is a rich source of mesenchymal stem cells and growth factors that promote angiogenesis, collagen synthesis, and extracellular matrix remodeling [6, 7]. Fat grafting has been explored in soft tissue regeneration, radiation injury repair, and pelvic floor reconstruction [8]. Its autologous and biocompatible nature suggests potential applicability as a biologically integrative adjunct for reinforcing weakened rectovaginal support while avoiding foreign materials.

In this context, we conducted a prospective pilot study to assess the feasibility and safety of autologous fat injection for rectocele repair and to explore short-term anatomical and functional outcomes. The primary objective was to evaluate changes in rectocele size on magnetic resonance (MR) defecography, while secondary objectives included exploratory assessment of obstructed defecation symptoms, defecation frequency, and clinical grading.

## Materials and methods

### Study design and setting

This was a prospective single-arm pilot study conducted between October 2023 and June 2025 at the Department of General Surgery, Kasr Al-Ainy Hospital, Cairo University. The study was designed to assess the feasibility, safety, and short-term anatomical and functional outcomes of autologous fat injection for rectocele repair.

### Ethical approval and trial registration

The study protocol was approved by the Cairo University Faculty of Medicine Research Ethics Committee (approval no. MD-260-2022) and was conducted in accordance with the principles of the Declaration of Helsinki (2013 revision). The trial was prospectively registered on ClinicalTrials.gov (NCT06604702: “Fat injection for rectocele treatment: a novel approach”). Trial registration was completed prior to enrollment of the first participant. Written informed consent was obtained from all patients before inclusion in the study.

### Patient selection

Women aged 18–70 years presenting with symptomatic rectocele associated with obstructed defecation syndrome (ODS) were screened for eligibility.

#### Inclusion criteria:

- Symptomatic rectocele classified as Baden–Walker grade II or III, confirmed by clinical examination and MR defecography.
- Presence of at least one symptom of obstructed defecation, including straining, incomplete evacuation, digital evacuation, or perineal support.

#### Exclusion criteria:

- Previous pelvic floor or pelvic organ prolapse surgery.
- Multicompartment pelvic organ prolapse requiring concomitant surgical procedures.
- Active anorectal pathology (e.g., fissure, fistula, abscess).
- Inflammatory bowel disease, pregnancy, or significant systemic comorbidity precluding anesthesia.

All consecutive eligible patients meeting these criteria during the study period were invited to participate. Although patients with Baden–Walker grade I rectocele were screened early during recruitment, all patients who proceeded to intervention met grade II–III criteria at baseline assessment and constituted the final study cohort. No eligible patients declined participation, and no patients were lost to follow-up during the minimum observation period.

### Preoperative assessment

Baseline patient data included age, body mass index (BMI), parity, comorbidities, and history of prior abdominal or pelvic surgery. All patients underwent clinical pelvic examination with grading of rectocele severity using the Baden–Walker halfway scoring system.

Preoperative imaging was performed using MR defecography to quantify rectocele size (measured in centimeters as maximal anterior rectal wall protrusion during straining). Obstructed defecation symptoms—including straining, incomplete evacuation, digital evacuation, and perineal support—were recorded using predefined clinical categories during routine assessment. Baseline defecation frequency was documented using categorical intervals.

Validated symptom or quality-of-life instruments were not employed, as this pilot study relied on predefined clinical symptom categories recorded during standard clinical evaluation.

### Operative technique

All procedures were performed by the same colorectal surgical team experienced in pelvic floor and minimally invasive reconstructive surgery.

#### Anesthesia and preparation

Patients were admitted on the day of surgery following bowel preparation consisting of a light diet and rectal enema on the preceding evening. Procedures were performed under spinal or general anesthesia according to anesthesiologist assessment. Sequential compression devices were applied for thromboprophylaxis, and a single intravenous dose of ceftriaxone (1 g) was administered 60 min prior to incision. The abdomen, perineum, and upper thighs were prepared and draped under sterile conditions.

#### Patient position for fat harvesting

Patients were initially positioned supine. Two 1–1.5 cm transverse skin incisions were made at the lateral ends of the lower abdominal crease within natural skin folds. A tumescent solution containing epinephrine (1 mg in 1000 mL normal saline; 1:1,000,000 dilution) was infiltrated into the subcutaneous fat using an 11-gauge blunt cannula. Subcutaneous fat was harvested by gentle manual liposuction using 20- and 50-mL Luer-lock syringes, generating low, controlled negative pressure. Cannula advancement was performed in a radial fan-shaped pattern within the deep subcutaneous plane to ensure even fat retrieval and minimize contour irregularities. The harvested lipoaspirate was collected in sterile syringes for immediate processing.

#### Fat processing

**Fig. 1:**
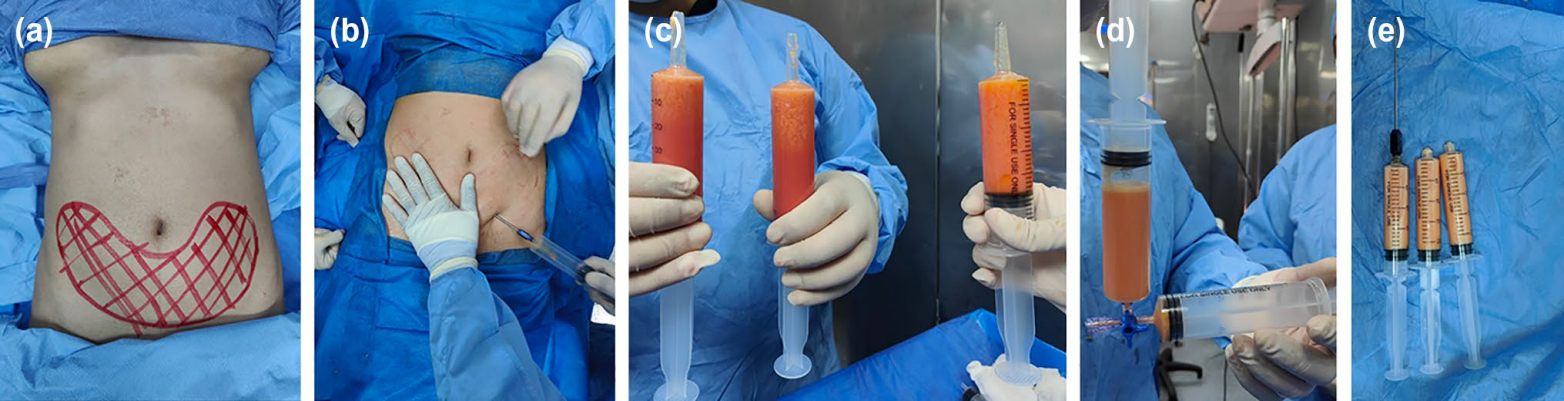
Fat harvesting and preparation (**A**–**E**)

The harvested fat was mechanically emulsified by repeated transfer (approximately 30–40 passes) between two 50-mL syringes connected via a sterile three-way stopcock until a homogeneous emulsion was obtained. The emulsified fat was filtered through sterile gauze to remove excess fluid, oil, and fibrous debris, then allowed to decant for 3–5 min. The supernatant was discarded, and the processed graft material was loaded into 20-mL syringes fitted with 15-gauge blunt injection cannulas (Fig. 1).

#### Patient repositioning and perineal preparation

**Fig. 2:**
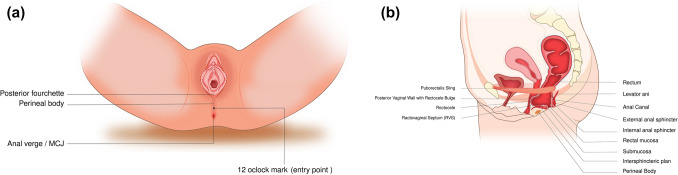
Anatomical orientation (**A**, **B**)

Following fat processing, patients were repositioned into the lithotomy position. The perineal and perianal regions were re-prepared and draped. A bimanual rectovaginal examination was performed to identify the rectocele pocket and delineate its cranial and lateral boundaries prior to injection (Fig. 2).

#### Injection technique

A 2–3 mm skin puncture was created at the 12 o’clock position approximately 2 cm superior to the posterior vaginal commissure. Through this entry point, a 15-gauge blunt cannula was advanced into the deep rectovaginal septal plane, anterior to the internal anal sphincter, under continuous digital vaginal guidance.

**Fig. 3:**
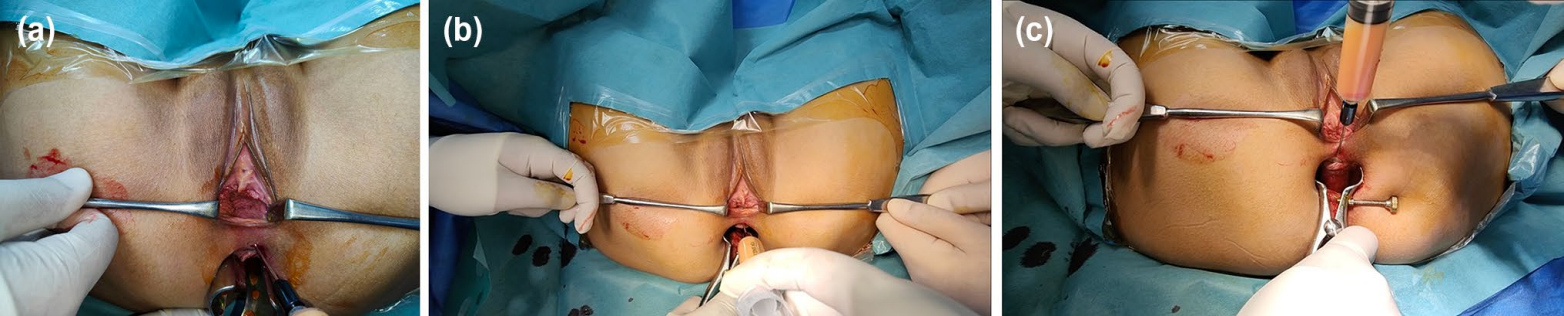
Injection technique (clinical photographs, **A**–**C**)

Autologous fat was injected slowly using a retrograde threading technique while withdrawing the cannula, depositing small aliquots in multiple passes to achieve uniform reinforcement of the rectovaginal septum. Injection was continued until the rectocele pocket was adequately obliterated and symmetric septal support was palpated both vaginally and rectally. The mean injected fat volume was 52 ± 10 mL (range 25–65 mL) (Fig. 3).

**Fig. 4:**
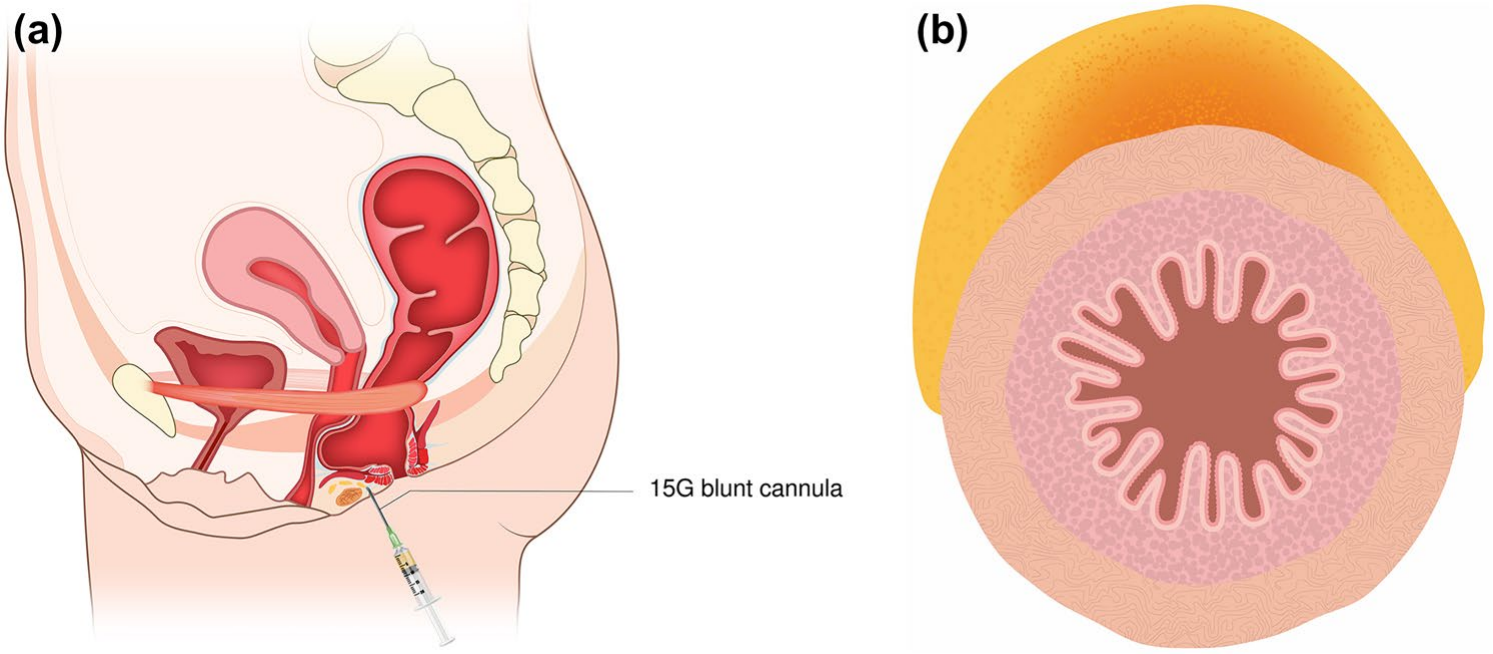
Injection pathway and fat distribution (**A**, **B**)

Care was taken to avoid violation of the internal or external anal sphincters. Cannula advancement and fat deposition were performed under continuous digital guidance to ensure correct depth and plane, and injection was limited to low-pressure retrograde delivery to minimize the risk of intravascular placement (Fig. 4).

**Fig. 5:**
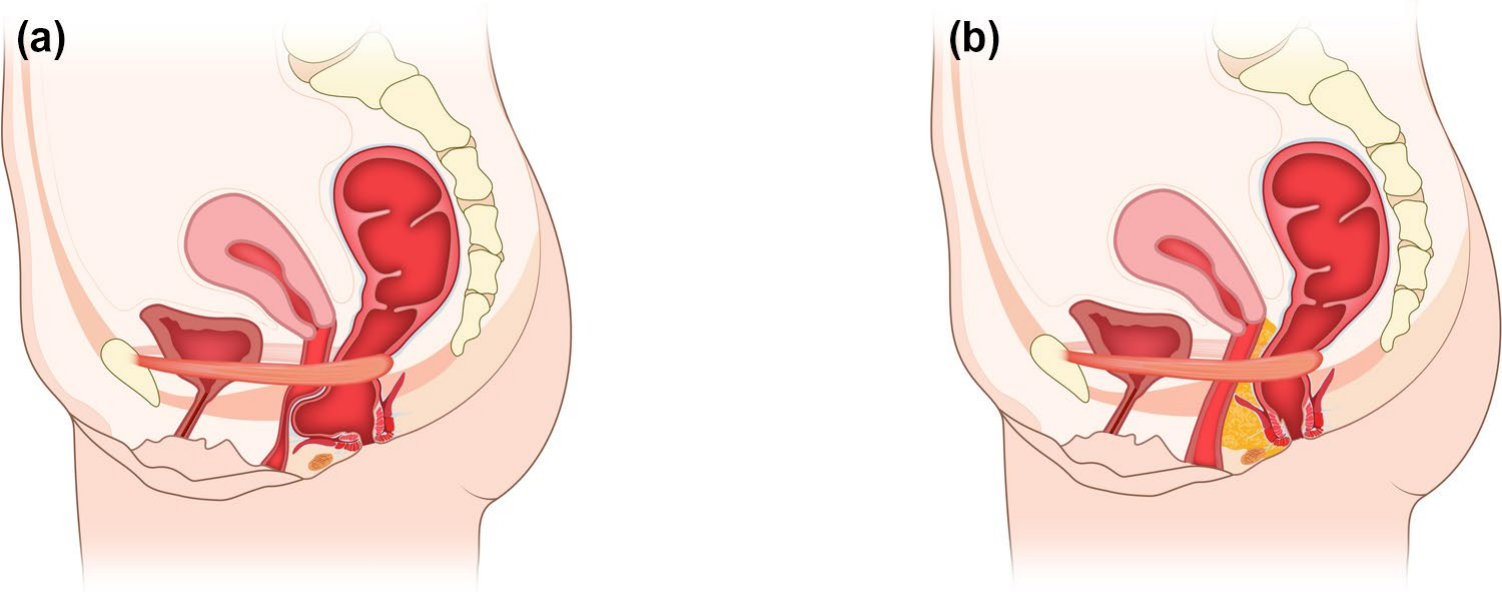
Pre- and post-injection anatomical outcome

The skin entry site was closed with 4-0 polyglactin (Vicryl) suture and covered with a sterile dressing (Fig. 5).

#### Immediate postoperative care

Patients were observed for 24 h and discharged the following day. Postoperative instructions included wearing an abdominal binder for 2 weeks, adherence to a high-fiber diet with stool softeners or osmotic laxatives for 4 weeks, and avoidance of strenuous activity and sexual intercourse for at least 2 weeks. Analgesia consisted of paracetamol with or without nonsteroidal anti-inflammatory drugs as required.

Postoperative continence was assessed clinically at each follow-up visit by structured questioning regarding new-onset fecal urgency, leakage, or soiling.

### Follow-up and outcome measures

Postoperative follow-up visits were scheduled at 2 weeks, 1 month, 3 months, 6 months, and 12 months, with subsequent visits every 6 months thereafter when feasible. Clinical follow-up duration varied according to patient availability, with a minimum follow-up of 6 months.

The primary outcome was change in rectocele size (cm) measured on MR defecography before and after intervention. Postoperative MR defecography was performed once for each patient at the final available follow-up visit (range 6–18 months), rather than at a fixed postoperative time point.

Secondary outcomes included change in clinical Baden–Walker grade, resolution of obstructed defecation symptoms (straining, incomplete evacuation, digital evacuation, and perineal support), change in defecation frequency categories, postoperative pain assessed using a visual analogue scale (VAS; 0–10), and procedure-related complications (including infection, constipation, recurrence, or need for re-intervention).

Patient-reported satisfaction was documented qualitatively during follow-up visits and is presented as exploratory data only. All functional and patient-reported outcomes were considered exploratory owing to the pilot nature of the study and the absence of validated symptom instruments.

### Statistical analysis

Statistical analysis was performed using IBM SPSS Statistics version 25 (IBM Corp., Armonk, NY, USA). Continuous variables were expressed as mean ± standard deviation (SD), and categorical variables as frequencies and percentages.

Pre- and postoperative continuous variables were compared using paired *t*-tests. Categorical paired data were analyzed using the McNemar test or the marginal homogeneity test, as appropriate. Normality of continuous variables was assessed visually and using the Shapiro–Wilk test prior to parametric analysis.

The relationship between injected fat volume and change in rectocele size was explored using Pearson’s correlation coefficient (*r*). Given the limited sample size, this analysis was considered exploratory.

No adjustment was made for multiple comparisons; therefore, all results should be interpreted cautiously. A two-sided *p*-value < 0.05 was considered statistically significant.

## Results

### Patient characteristics

Between October 2023 and June 2025, 25 women with symptomatic rectocele and obstructed defecation syndrome (ODS) were included in the study. The mean age was 35 ± 6 years (range 25–45), and the mean body mass index was 28 ± 2 kg/m^2^. All patients were multiparous (median parity 3, range 1–5) and had Baden–Walker grade II–III rectocele at baseline. No patient had a history of prior pelvic floor or prolapse surgery or significant comorbid disease. Baseline demographic and clinical characteristics are summarized in Table 1.

**Table 1:** Baseline patient demographics and characteristics

Variable	Mean ± SD/*n* (%)
Number of patients	25
Age (years)	35 ± 6 (25–45)
Body mass index (kg/m^2^)	28 ± 2 (25–31)
Parity (median, range)	3 (1–5)
Previous pelvic surgery	0 (0%)
Baden–Walker grade II–III	25 (100%)
Straining	25 (100%)
Incomplete evacuation	25 (100%)
Digital evacuation	25 (100%)
Perineal support	25 (100%)
Mean rectocele size (cm)	3.28 ± 0.82
Defecation frequency ≥ 3–4 days between bowel movements	25 (100%)
Comorbidities (hypertension/diabetes mellitus [DM])	0 (0%)

### Operative data

All procedures were completed successfully without intraoperative complications. The mean operative time was 45 ± 3 min. The mean volume of harvested fat was 150 ± 25 mL, and the mean injected volume was 52 ± 10 mL (range 25–65 mL). All patients were discharged within 24 h. Operative and perioperative details are summarized in Table 2.

**Table 2:** Operative details and perioperative findings

Parameter	Value (mean ± SD, median [range], or *n* [%])
Anesthesia	Spinal: 19 (76%); general: 6 (24%)
Operative time (min)	45 ± 3
Harvested fat (mL)	150 ± 25
Injected fat (mL)	52 ± 10 (25–65)
Intraoperative complications	0 (0%)
Hospital stay (days)	1 ± 0
Early postoperative pain (VAS 0–10)	5 ± 1 (3–7)
Transient constipation	5 (20%)
Recurrence of ODS symptoms	5 (20%)
Redo injection required	2 (8%)
Infection/hematoma/seroma	0 (0%)
Mean follow-up (months)	11 ± 4 (6–18)
Patient-reported satisfaction *(verbal)*	25 (100%) reported subjective satisfaction

### Postoperative course and complications

Postoperative recovery was uneventful in all patients. Mean pain scores (VAS 0–10) were 5 ± 1 on postoperative day 1 and decreased thereafter. No cases of infection, hematoma, seroma, donor-site irregularity, or pelvic sepsis were observed.

Transient postoperative constipation occurred in five patients (20%) and resolved with conservative management within 2 weeks. Recurrence of obstructed defecation symptoms was observed in five patients (20%) during follow-up. Two patients (8%) underwent repeat fat injection using the same protocol, with subsequent symptomatic improvement. No patient developed new-onset fecal incontinence, urgency, or dyspareunia.

The mean clinical follow-up duration was 11 ± 4 months (range 6–18 months).

### Functional outcomes

Marked improvement in obstructed defecation symptoms was observed following intervention. Preoperatively, all patients reported straining, incomplete evacuation, digital evacuation, and perineal support. Postoperatively, these symptoms decreased significantly, with straining, incomplete evacuation, and digital evacuation reported by 20% of patients, and no patients reporting the need for perineal support (*p* < 0.001 for all comparisons).

Defecation frequency also improved significantly. At final follow-up, 60% of patients reported daily bowel movements, 20% reported bowel movements every other day, and 20% reported occasional delay (*p* < 0.001).

Patient-reported satisfaction was documented qualitatively during follow-up visits and is presented as exploratory data only.

### Anatomical outcomes

Clinical examination demonstrated significant improvement in rectocele severity. While all patients had Baden–Walker grade II–III rectocele preoperatively, 68% were graded as stage 0–I at final follow-up (*p* < 0.001, marginal homogeneity test).

MR defecography demonstrated a significant reduction in rectocele size from a preoperative mean of 3.28 ± 0.82 cm to a postoperative mean of 1.82 ± 0.47 cm (mean reduction 1.46 cm; *p* < 0.001, paired *t*-test).

No cases of postoperative worsening or development of new pelvic organ prolapse were observed.

### Exploratory volume–outcome analysis

**Fig. 6:**
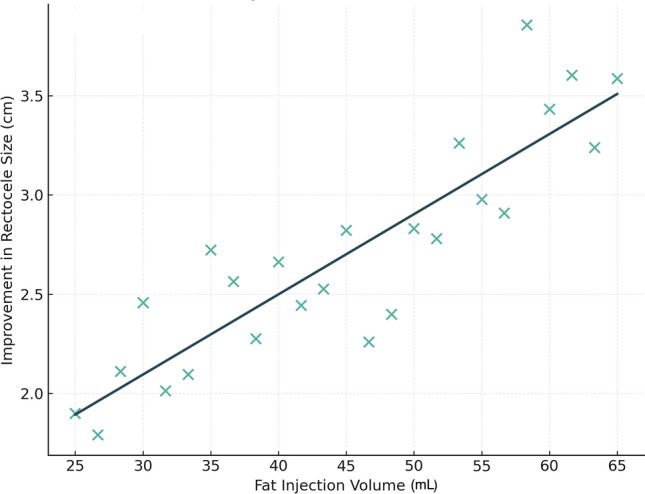
Scatter plot demonstrating the exploratory association between injected fat volume and reduction in rectocele size. Each point represents an individual patient. A moderate positive correlation was observed (Pearson r = 0.435, p = 0.030); however, this analysis was exploratory and not powered to define optimal dosing or volume thresholds.

An exploratory correlation analysis demonstrated a moderate positive association between injected fat volume and reduction in rectocele size (Pearson *r* = 0.435, *p* = 0.030) (Fig. 6). Patients receiving larger graft volumes tended to demonstrate greater anatomical improvement; however, given the limited sample size and absence of adjustment for multiple comparisons, this finding should be interpreted cautiously.

## Discussion

This prospective pilot study suggests that autologous fat injection into the rectovaginal septum is a feasible and safe minimally invasive approach for selected patients with symptomatic rectocele. The intervention was associated with short-term anatomical improvement on MR defecography and parallel improvement in obstructed defecation symptoms, with minimal morbidity and no observed continence impairment. Given the single-arm design and exploratory outcome measures, these findings should be interpreted as preliminary and hypothesis-generating.

Surgical management of posterior compartment prolapse remains challenging, with existing techniques offering variable durability and functional outcomes. Posterior colporrhaphy and site-specific rectocele repair are widely practiced but have been associated with recurrence rates that vary substantially across series. Mesh-reinforced repairs may improve anatomical outcomes in selected cases but carry well-recognized risks of mesh-related complications. Minimally invasive approaches such as stapled transanal rectal resection have demonstrated symptomatic benefit in obstructed defecation but are associated with procedure-specific morbidity. Collectively, these limitations have driven continued interest in alternative strategies that aim to reinforce posterior compartment support while minimizing surgical trauma and foreign material implantation.

The rationale for autologous fat injection lies in both its volumetric and biological properties. Adipose tissue contains adipose-derived stem cells and a stromal vascular fraction rich in angiogenic and immunomodulatory mediators that may promote tissue remodeling and extracellular matrix reinforcement. While fat grafting has been explored in various reconstructive and regenerative contexts, evidence supporting its application in pelvic floor disorders remains limited. The present study contributes early clinical data suggesting that biologically integrative reinforcement of the rectovaginal septum may be associated with measurable anatomical and functional improvement.

Importantly, no patient in this series developed new-onset fecal incontinence, urgency, or dyspareunia. Particular attention was paid to maintaining injection within the deep rectovaginal septal plane anterior to the internal anal sphincter, with continuous digital guidance used to avoid sphincter violation. These findings provide preliminary reassurance regarding procedural safety; however, formal continence scoring and longer-term follow-up will be required to fully characterize functional outcomes.

An exploratory association between injected fat volume and anatomical improvement was observed. This finding should be interpreted cautiously, as the study was not powered to define dose–response relationships or establish volume thresholds. Observed volume-related trends are best viewed as descriptive observations that may inform the design of future trials rather than prescriptive guidance for clinical practice.

Several limitations warrant emphasis. The study was limited by its small sample size, single-arm design, and absence of a comparator group, precluding causal inference or comparison with established surgical techniques. Functional outcomes were assessed using predefined clinical categories rather than validated symptom or quality-of-life instruments, and neither patients nor assessors were blinded. Postoperative MR defecography was performed at variable time points corresponding to the final available follow-up visit, introducing potential temporal variability in anatomical assessment. Together, these factors underscore the exploratory nature of the findings.

Despite these limitations, this pilot study demonstrates that autologous fat injection for rectocele repair is technically feasible, well tolerated, and associated with short-term anatomical and functional improvement. These results support further investigation in larger, comparative studies incorporating standardized imaging time points, validated patient-reported outcome measures, and longer-term follow-up to define the durability and clinical role of this technique within contemporary pelvic floor surgery.

## Conclusions

Autologous fat injection into the rectovaginal septum appears to be a feasible and safe minimally invasive approach for selected patients with symptomatic rectocele. In this prospective pilot study, the technique was associated with short-term anatomical improvement on MR defecography and parallel improvement in obstructed defecation symptoms, with minimal morbidity and no observed impairment of continence.

Given the single-arm design, small sample size, and exploratory outcome measures, these findings should be interpreted as preliminary. Larger comparative studies incorporating validated patient-reported outcomes, standardized imaging time points, and longer-term follow-up are required to define the durability and clinical role of autologous fat injection within contemporary rectocele management.

## Data Availability

The datasets generated and analyzed during the current study are available from the corresponding author on reasonable request.

## Declarations

### Clinical trial registration

The trial was prospectively registered on ClinicalTrials.gov under the identifier NCT06604702 (“Fat injection for rectocele treatment: a novel approach”).
